# Effect on Microstructure and Mechanical Properties of Microwave-Assisted Sintered H13 Steel Powder with Different Vanadium Contents

**DOI:** 10.3390/ma15041273

**Published:** 2022-02-09

**Authors:** Xuebin Chen, Lei Zhao, Min Wei, Danqi Huang, Liwu Jiang, Haizhou Wang

**Affiliations:** 1 Beijing Advanced Innovation Center for Materials Genome Engineering, National Center for Materials Service Safety, University of Science and Technology Beijing, Beijing 100083, China; xbinchen@yeah.net; 2 Beijing Key Laboratory of Metal Materials Characterization, The NCS Testing Technology Co., Ltd., Beijing 100081, China; zhaolei@ncschina.com (L.Z.); B20200484@xs.ustb.edu.cn (M.W.); huangdanqi@ncschina.com (D.H.)

**Keywords:** microwave sintering metals and alloys, microstructure, powder technology, microhardness, wear and tribology

## Abstract

The present work demonstrated the first-ever preparation of block specimens by the microwave sintering of H13 alloy powder. Varying proportions of vanadium powder (1.5%, 2.5%, 3.5%, 4.5%, and 5.5% on a mass basis) were added to H13 mold steel and these mixtures were sintered using microwaves. X-ray fluorescence spectroscopy was employed to determine the compositions of the resulting specimens and vanadium percentages of 1.56%, 2.04%, 3.10%, 4.06%, and 4.20% were determined. These results demonstrate a clear trend, with significantly lower vanadium amounts than expected based on the nominal values at higher vanadium loadings. Different samples were also found to exhibit different degrees of ablation, and this effect was related to the presence of voids in the materials. The surface compositions of these specimens were examined by laser-induced breakdown spectroscopy and were found to be relatively uniform. The microstructures as well as the hardness properties of the materials were assessed. Microwave sintering of 100 g specimens at 1300 °C for 10-min generated samples with hardness values ranging from 205 HV (at the lowest vanadium content) to 175.2 HV (at the highest vanadium content). The wear behavior of samples prepared by microwave sintering H13 die steel with different vanadium contents at room temperature has been studied. The results showed that 1.5% vanadium content is the best mass ratio.

## 1. Introduction

Microwaves provide rapid heating that shortens the time and temperature required to sinter various materials, thus simplifying preparation processes. Consequently, microwave heating has become an experimental tool used to examine the relationships between material composition, phase, structure, and properties, and has been applied to the discovery and basic research of new materials [1,2,3,4]. Metals are good conductors and do not absorb microwaves, so metal utensils cannot normally be placed in a standard domestic microwave oven. However, Roy et al. demonstrated that metal powders act as good microwave absorbers and successfully used microwave sintering to fabricate items such as small gears [4]. Since that initial work, there has been widespread interest in using microwaves to process metallic materials [5,6,7,8,9,10,11]. As an example, Leparoux et al. used SiC to enhance the sintering of Al matrix composites in conjunction with the application of microwave energy, and found that both the microwave absorption and heating rate increased with decreases in the SiC particle size [12]. Since the 1990s, the application of microwaves to the sintering of various materials has expanded. Zhou et al. examined the microwave sintering of a WC-Co fine cemented carbide and compared the results to those obtained with conventional sintering processes [13]. The results showed that the flexural strength, hardness, and magnetic force of the samples were all greatly improved after microwave sintering.

H13 die steel (4Cr5MoSiV1) exhibits thermal stability and thermal fatigue resistance along with high strength, toughness, and excellent wear resistance [14,15,16,17,18]. In recent years, many researchers have improved the performance of H13 die steel by adding microalloying elements such as niobium, tungsten, aluminum, and rare earth metals [19,20,21,22,23,24]. When the mass fraction of vanadium was 0.105%, the MX phase was diffusely distributed in the matrix, and now the phase owned the largest number and smallest size. The tensile strength of the tested steel increased from 325.2 Mpa to 289.4 Mpa, and the creep-rupture life decreased from 370 h to 251 h when the mass fraction of vanadium increased from 0.105 wt.% to 0.203 wt.%. Yang et al. [25] found that the incorporation of 0.105% vanadium by mass generating a finely dispersed second phase had an 11Cr-2W steel structure within the primary matrix that promoted precipitation strengthening. Liu et al. [26] also reported that adding vanadium to medium chromium white cast iron refines the structure and improves the strength and wear resistance. Li et al. [27]. The results show that the amount of carbide in the as-cast specimens increases with the increase of vanadium content, the sample containing 0.4% vanadium has the best mechanical properties. After heat treatment, the microstructure of the sample is composed of residual austenite, lower bainite, and 10% carbide. Its tensile strength is 1070 MPa, hardness is HRC 52.9, impact toughness is 28.26 J/cm^2^, and wear rate is 0.54 mg/m. Relative wear resistance is increased by 24% than that of samples without vanadium. Ma et al. [28].

In this study, we investigated the effects of adding various amounts of vanadium to H13 metal powder in combination with the use of a microwave sample preparation process. The compositional changes were analyzed to ensure a suitable degree of compositional uniformity. The organizational state and friction and wear properties of the various specimens were investigated as part of a preliminary investigation of the effect of compositional variation. The main purpose was to study the effect of microwaves on the composition, microstructure, and properties of bulk materials prepared by microwave sintering alloy powders.

## 2. Materials and Methods

H13 metal powder (average particle size: 20 μm) (Flance, Beijing, China) was used as the parent matrix together with vanadium powder (average particle size: 20 μm, purity > 99.9%) (Flance, Beijing, China) as the additive. The chemical composition of the H13 powder is provided in Table 1. The vanadium powder was added at mass-based proportions of V1: 1.5%, V2: 2.5%, V3: 3.5%, V4: 4.5%, and V5: 5.5%, after which each mixture was milled in a planetary ball mill (MITR, Changsha, China), employing steel balls with diameters of 5–10 mm, a ball-to-powder mass ratio of 6:1, a rotational rate of 200 rpm and a milling time of 6 h. After mechanical alloying, each specimen was pre-compressed at 300 MPa for 1 min. Finally, each metal compact ball was placed in the microwave oven for sintering.

**Figure 1:**
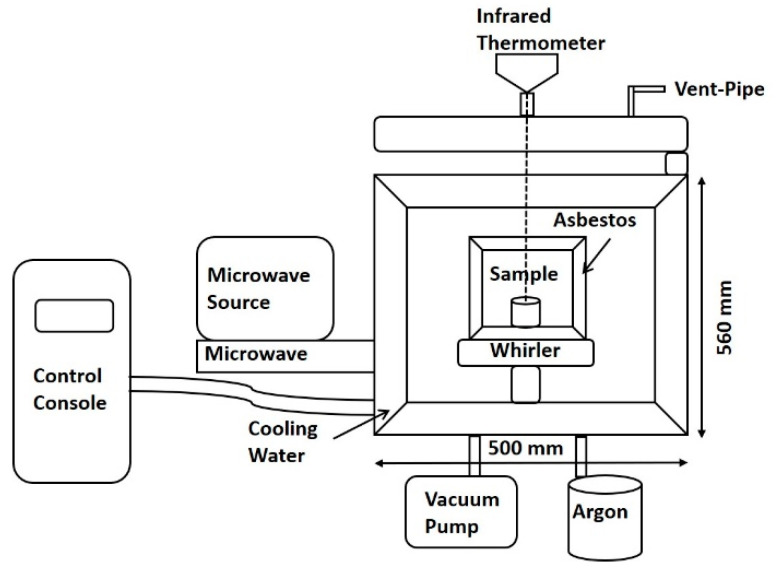
Schematic diagram of the microwave apparatus.

The microwave heating device used in this work (fabricated at the Kunming University of Science and Technology, WSP-2450-5000, Kunming, China) is shown in Figure 1. This apparatus applied a microwave frequency of 2.45 GHz and a power of 5.5 kW. An alumina (Al_2_O_3_) crucible was used in these experiments, with asbestos fibers as insulation and an infrared thermometer (Raytek MM2MH, Santa Cruz, CA, USA). The detection range of 300 to 1600 °C and the probe head had an emissivity value of 0.75.

**Figure 2:**
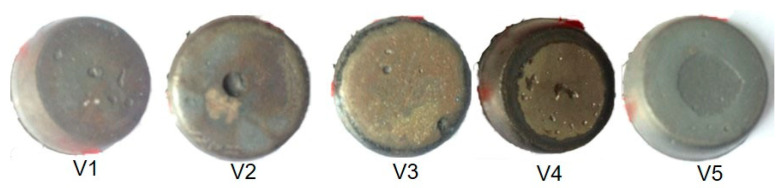
Schematic diagram of microwave preparation of gradient samples.

**Figure 3:**
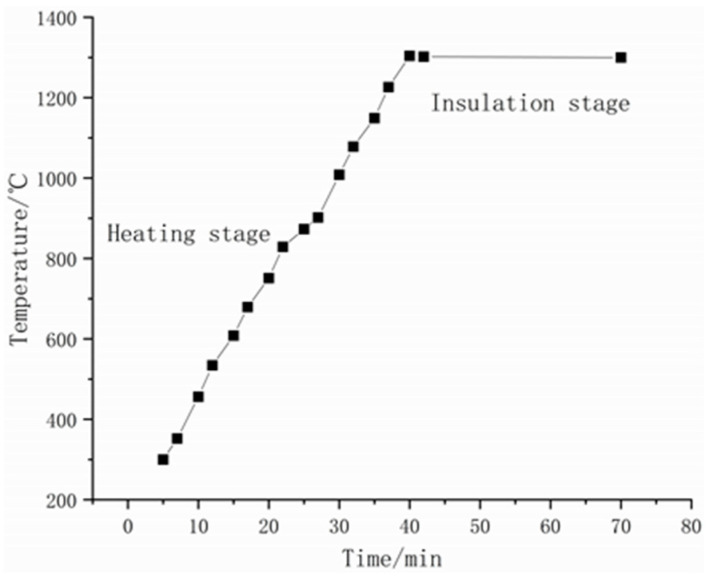
The sintering profile of sample preparation.

Prior to microwave sintering, after each sample was placed in the microwave oven, the oven was evacuated to 0.4 MPa, then filled with argon gas to atmospheric pressure. This step was repeated three times to ensure that as much oxygen as possible was removed from the oven. Throughout the entire sintering process, the oven temperature was controlled by manually entering the heating program such that the sample heating rate was maintained at 30 °C/min. The sintering was performed under a flow of 99.9% argon, with the specimen held at 1300 °C for 30 min. After sintering, the sample was allowed to naturally cool to ambient temperature while remaining in the furnace. The sintered sample is shown in Figure 2. The sintering profile is shown in Figure 3. 

Each sample was cut into cubic specimens with a wire cutter (15 mm × 10 mm × 5 mm). After grinding, polishing, and immersion in a 4% (by volume) alcohol nitrate solution (Concentrated nitric acid, concentration 65%, analytical grade; Sinopharm, Beijing, China), the microstructures of these cubes were observed by optical microscopy (OM) using a LEICA dm-6000 m instrument (Wetzlar, Germany). Samples were also assessed after sintering using a microbeam X-ray fluorescence (XRF) spectrometer (M4 Tornado, Bruker, Rheinstetten, Germany), employing a rhodium X-ray tube, a silicon drift detector with an effective area of 30 mm^2^, and a tube voltage and current value of 40 kV and 200 μA, respectively. Laser-induced breakdown spectroscopy (LIBS) using an OPA 200 instrument was performed at the NCS Testing Technology Co., Ltd., Beijing, China, with an Nd: YAG pumped solid-state laser (wavelength = 1064 nm, laser pulse rate = 10 Hz, spot size = 250 μm, pump voltage = 1.35 kV) to characterize the surface composition distribution of each sample (based on two pre-denudation and two denudation tests). The morphology of each specimen and the presence of surface precipitates were determined using tungsten filament scanning electron microscopy (SEM; JSM IT-300, JEOL, Tokyo, Japan, 20 kV, 1.5 nA, backscattered electron detector) combined with energy-dispersive X-ray spectroscopy (EDS; Azetec max50, Oxford, UK, spot size = 210 nm, dwell time = 32 μs/pxl). Micro-hardness data were acquired with a Qness q-10 tester (pressure = 0.5 kg, compress time = 15 s; Qness, Vienna, Austria). A multifunctional friction and wear testing machine (MFT-5000, Rtec Instruments, San Jose, CA, USA) was used to test the wear properties of each sample in reciprocating friction mode using a Si_3_N_4_ grinding ball with a diameter of 6 mm to slide the sample for 30 min at a sliding distance of 5 mm under the conditions of 50 N and 3 Hz. The total volume of the wear track was measured by three-dimensional (3D) measuring laser microscopy (OLS4100, OLYMPUS, Tokyo, Japan).

## 3. Results

### 3.1. Distributions of Vanadium in Samples

XRF was used to ascertain the elemental composition of each specimen. XRF does not provide as strong a signal for vanadium as atomic emission spectroscopy but generates more accurate data [29]. The vanadium content of each specimen is provided in Figure 2.

**Figure 4:**
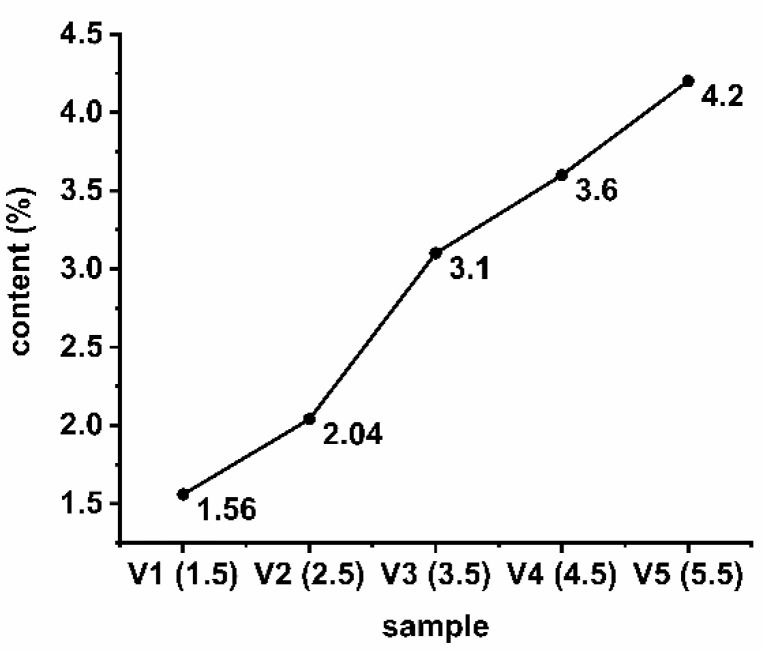
Vanadium content in each sample, as determined by XRF.

As shown in Figure 4, the data confirm an obvious gradient in these samples with regard to the vanadium content. It is evident from these data that the prepared samples had vanadium concentrations below the nominal values in the original powder mixtures. Even so, the surface of the sintered powder retained some oxygen that reacted with the vanadium to form an oxide residue during sintering, which reduced the vanadium content. In addition, the degree of sample ablation that occurred during the sintering process, which reduced the amount of vanadium, varied between samples.

**Figure 5:**
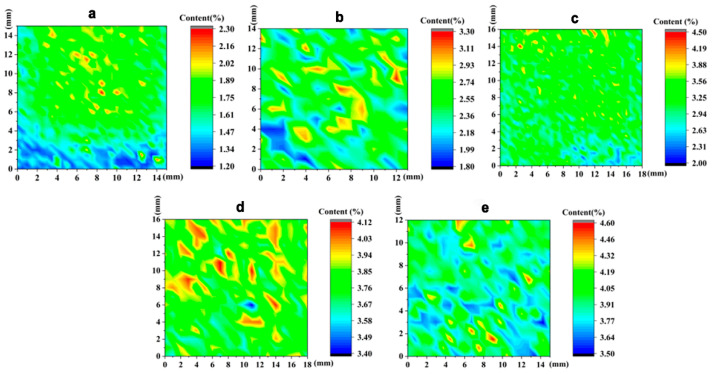
Distributions of vanadium on the sample surfaces (**a**: V1; **b**: V2; **c**: V3; **d**: V4; **e**: V5).

The distribution of vanadium within a 15 × 15- mm area on each sample surface was characterized using LIBS, as shown in Figure 5. These data demonstrate that the vanadium distributions on the sample surfaces were relatively uniform, albeit with some degree of segregation in certain areas. Chen et al. [30] studied the microwave preparation of H13 die steel specimens having different tungsten proportions and found that the presence of pores on the sample surfaces after sintering also affected the measurement results.

### 3.2. Effects of Vanadium on Microstructure

**Figure 6:**
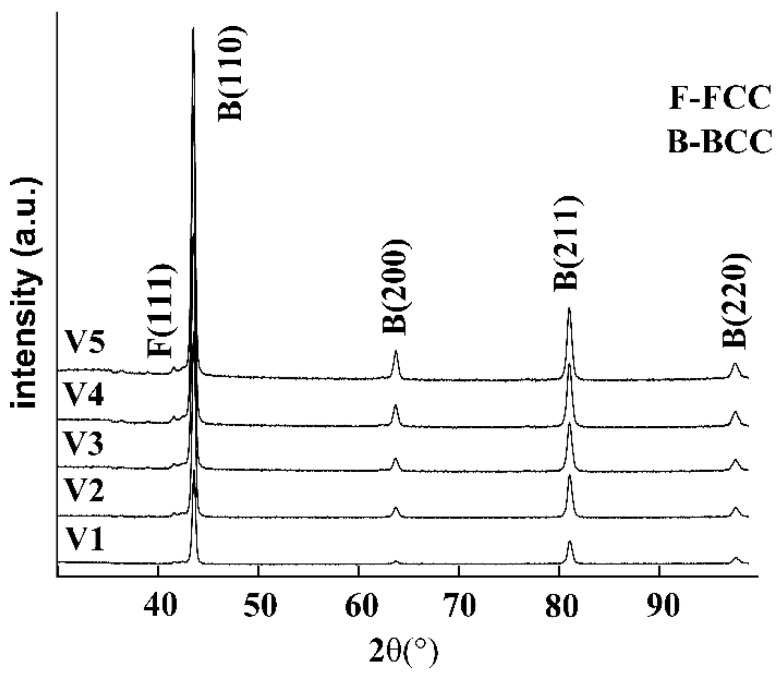
XRD patterns of different vanadium content.

XRD analysis of samples with different V contents is shown in Figure 6, it is apparent that the sample has a VC (111) characteristic peak of FCC phase structure; it can be concluded that with the increase of V content, the intensity of (111) diffraction peak also increases.

**Figure 7:**
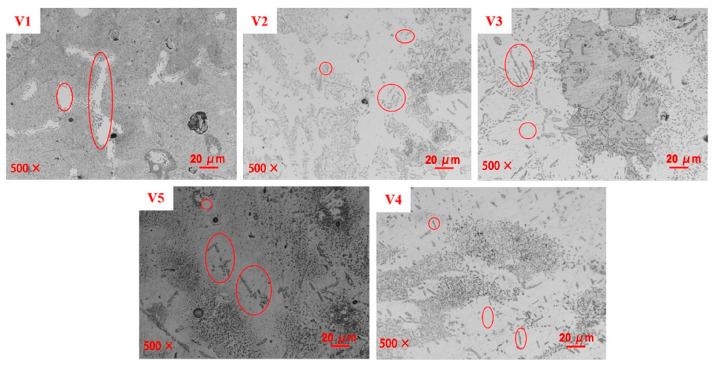
Effects of vanadium content on microstructure.

The microstructures of the samples as observed through a metallurgical microscope are shown in Figure 7. Precipitates are seen on the surfaces and, as the vanadium content was increased, the size of these precipitates gradually increased while the quantity decreased. Based on the smaller precipitate sizes in sample V1 compared with the less concentrated but larger particles on sample V5, it can be concluded that the size of these precipitates was positively correlated with the vanadium content. Additionally, the number of precipitates was negatively correlated with the proportion of vanadium.

**Figure 8:**
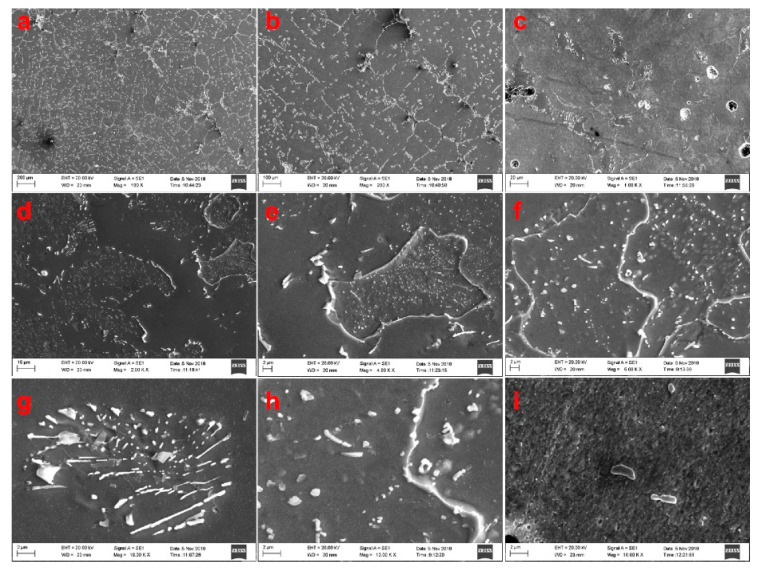
SEM images showing the effects of vanadium content on microstructure (**a**: 100×; **b**: 200×; **c**: 1000×; **d**:2000×; **e**,**f**: 5000×; **g**–**i**: 10,000×).

These precipitates were also observed using scanning electron microscopy, as shown in Figure 8. A large number of fishbone-like precipitates are clearly seen on the sample surfaces at low magnifications. With increased magnification, it is apparent that the grid-like distribution on these specimens resulted from greater quantities of precipitates. At high magnification, both slender fishbone-like precipitates and granular precipitates are observed. The former surrounded the latter, and rod-shaped precipitates with intermediate sizes are also seen to have been scattered over the sample surfaces.

**Figure 9:**
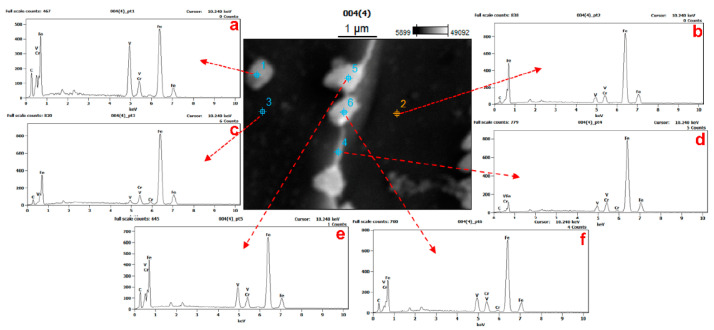
Energy spectra of samples having various vanadium proportions (**a**: pt1; **b**: pt2; **c**: pt3; **d**: pt4; **e**: pt5; **f**: pt6).

The precipitates observed in SEM images (EHT = 20 kV, WD = 20 mm) were also evaluated by acquiring energy spectra from selected fishbone and granular precipitates as well as bulk regions to characterize the surface distribution of vanadium, with the results shown in Figure 9. The regional surface distributions indicate that the precipitates were enriched with vanadium.

It is also apparent that the proportions of vanadium and carbon at the precipitate corresponding to point pt1 were significantly higher than those at the bulk regions corresponding to points pt2 and pt3, while point pt4 was intermediate between the two precipitates. There was little variation in the elemental composition within the body of the sample, while the precipitates corresponding to points 5 and 6 showed higher carbon and vanadium contents than the surrounding material. On this basis, it can be concluded that the precipitates were made of vanadium carbide. The data in Table 2 demonstrate that the elemental composition at point 3 was similar to that at point 2, while point 4 (which was a link to the precipitate area) was not greatly different from the bulk material.

### 3.3. Effects of Vanadium on Hardness

**Figure 10:**
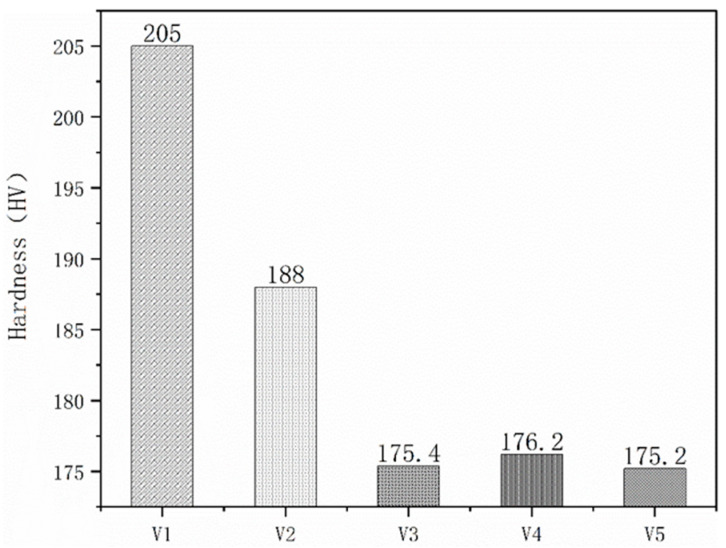
Microhardness values of the different specimens.

The effect of the vanadium content on the hardness of the samples was assessed by randomly taking five hardness points in the sample and then taking the average value. This process allowed the calculation of average hardness values for the samples, which were believed to be more useful for evaluation purposes when exploring changes in the overall hardness of the H13 die steel with increasing vanadium content. The results are summarized in Figure 10. These data confirm that the microhardness gradually decreased with increases in the vanadium content. When the vanadium content was in the range of 1.5–3.5%, the microhardness gradually decreased with increasing vanadium content. The microhardness tended to be stable when vanadium content was in the range of 3.5–5.5%. This effect is attributed to the gradual decrease in the number of vanadium carbide precipitates as the size of the precipitates increased.

### 3.4. Effects of Vanadium on Friction and Wear

**Figure 11:**
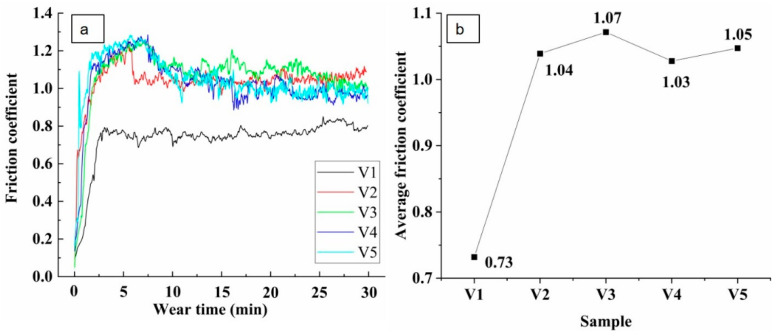
(**a**): Friction coefficients and (**b**): average friction coefficients of the samples.

The friction coefficients of the samples with different vanadium contents are shown in Figure 11a. In the early wear period (<7 min), the friction coefficients of the samples all greatly changed. This is because in the early wear running-in stage, the smooth grinding surface of the grinding ball under pressure suddenly becomes rough, resulting in a sudden increase in the friction coefficient. The friction coefficient of V1 greatly varied within the first 3 min, which indicates that the wear surface was continuously roughened during the wear process, and samples V2–V5 were rougher than V1, the effect of reducing the growth of the friction coefficient after about 2 min, then a drop after about 6–8 min and then reaching a quasi-stable mode. The average friction coefficients are shown in Figure 11b. The average friction coefficient of V1 was 0.73, while the average friction coefficient of V2–V5 was 1.04, 1.07, 1.03, and 1.05, respectively. This is because when higher content of vanadium is added, the vanadium particles do not form a continuous structure with the matrix alloy powder, which makes damage during the wear process easier and more wear chips appear, which aggravate the surface roughness, resulting in improvement of the surface friction coefficient of the material.

**Figure 12:**
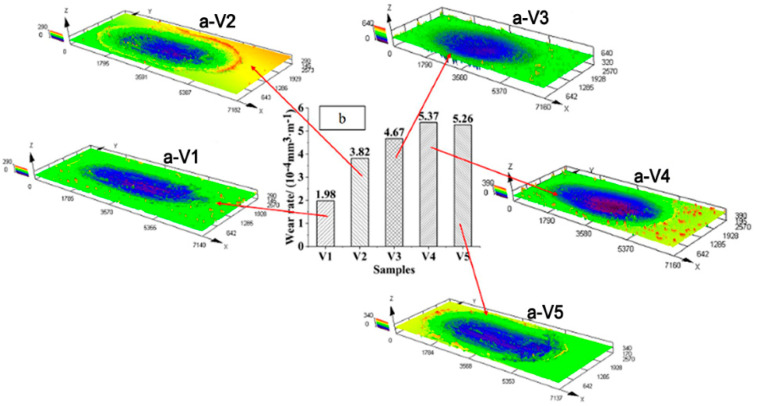
(**a**) (V1–V5) 3D outlines and (**b**): wear rates of the samples.

The 3D contour maps of the worn parts of the samples with different vanadium contents are shown in Figure 12a. The wear mark width of V4 was 30% larger than that of V1. The wear rates of the samples are shown in Figure 12b. The wear rate of V4 was 63.1% higher than that of V1. This is because the surface roughness of the sample increased with increasing vanadium content during the friction process, and third-body abrasive particles formed under extrusion wear by the reciprocating load, which increased the wear rate and the width of the wear mark on the sample.

**Figure 13:**
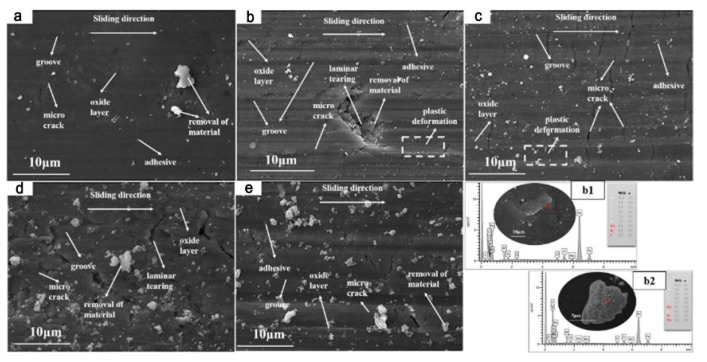
(**a**: V1; **b**: V2; **c**: V3; **d**: V4; **e**: V5) Morphologies of the worn surfaces of the samples. (**b1**,**b2**) Spectral comparison of the matrix and exfoliation, respectively.

The wear-surface morphologies of the samples are shown in Figure 13a–e. There were many small grooves along the sliding direction on the worn surfaces. With increasing vanadium content, the number of peeling particles, cracks, and deformation caused by extrusion wear on the V1 surface clearly increased. This phenomenon was verified by the gradual increase of the friction coefficient. The increase of the number of peeling particles on the sample surface increases the friction coefficient, which makes it easier to destroy the sample surface and produce more cracks and plastic deformation [31]. The sample surface energy spectra are shown in Figure 13b. By comparing the matrix (Figure 13(b1)) with the exfoliated particle (Figure 13(b2)), the increase of the oxygen content in the exfoliated particles during the wear process indicates that a large number of exfoliated oxide particles formed on the surface of the sample during the friction extrusion process, and the number of exfoliated oxide particles was positively correlated with the vanadium content.

## 4. Conclusions

Microwave sintering was used to prepare five specimens from H13 metal powders having different vanadium contents. The vanadium in these samples was found to be evenly distributed. Because the metal powder mixtures contained residual surface oxygen, vanadium oxides were generated during sintering, reducing the vanadium content. At the same time, different degrees of ablation occurred, which also reduced the amount of vanadium in the specimens after sintering. Vanadium carbide precipitates were identified on the sample surfaces after adding different proportions of vanadium. With increases in the vanadium content, the number of such precipitates gradually decreased but the size of the precipitates increased. The microhardness was found to gradually decrease with increases in the vanadium content because of changes in the number of vanadium carbide precipitates. When the vanadium content was in the range of 1.5–3.5%, the microhardness gradually decreased with increasing vanadium content. The microhardness tended to be stable when vanadium content was in the range of 3.5–5.5%. With increasing vanadium content, the friction coefficient of the sample increased. The effect of reducing the growth of the friction coefficient after about 2 min, then a drop after about 6–8 min, and then reaching a quasi-stable mode. This is because the number of exfoliated particles on the sample surface increased, resulting in the formation of third-body abrasive particles, which increased the surface roughness and led to an improvement of the friction coefficient of the material surface.

## Figures and Tables

**Table 1:** Elemental composition of the H13 metal powder.

Sample			Elementary Composition (%)		
	C	Si	Mn	P	S
	0.34	1.17	0.5	0.011	0.0073
H13	Cr	Ni	Cu	Mo	V
	5	0.098	0.032	1.16	1
	As	W			
	0.0026	<0.1			

**Table 2:** Element compositions determined from energy spectra at various points.

Location	C	V	Cr	Fe
pt1	11.17	24.86	3.92	60.05
pt2	2.50	4.15	5.03	88.32
pt3	3.03	1.92	5.79	89.26
pt4	1.29	3.70	5.39	89.62
pt5	9.59	11.47	4.74	74.20
pt6	6.08	8.45	5.68	79.80

## Data Availability

All datasets generated for this study are included in the manuscript.
